# Effect of Anodal-tDCS on Event-Related Potentials: A Controlled Study

**DOI:** 10.1155/2016/1584947

**Published:** 2016-11-10

**Authors:** Ahmed Izzidien, Sriharasha Ramaraju, Mohammed Ali Roula, Peter W. McCarthy

**Affiliations:** ^1^Social Cognition Laboratory, Harvard University, Cambridge, MA, USA; ^2^Medical Electronics and Signal Processing Research Unit, University of South Wales, Treforest, UK; ^3^Clinical Technology and Diagnostics Research Unit, University of South Wales, Treforest, UK

## Abstract

We aim to measure the postintervention effects of A-tDCS (anodal-tDCS) on brain potentials commonly used in BCI applications, namely, Event-Related Desynchronization (ERD), Event-Related Synchronization (ERS), and P300. Ten subjects were given sham and 1.5 mA A-tDCS for 15 minutes on two separate experiments in a double-blind, randomized order. Postintervention EEG was recorded while subjects were asked to perform a spelling task based on the “oddball paradigm” while P300 power was measured. Additionally, ERD and ERS were measured while subjects performed mental motor imagery tasks. ANOVA results showed that the absolute P300 power exhibited a statistically significant difference between sham and A-tDCS when measured over channel Pz (*p* = 0.0002). However, the difference in ERD and ERS power was found to be statistically insignificant, in controversion of the the mainstay of the litrature on the subject. The outcomes confirm the possible postintervention effect of tDCS on the P300 response. Heightening P300 response using A-tDCS may help improve the accuracy of P300 spellers for neurologically impaired subjects. Additionally, it may help the development of neurorehabilitation methods targeting the parietal lobe.

## 1. Introduction

Transcranial Direct Current Stimulation (tDCS) is a noninvasive brain stimulation technique which has recently gained interest in neuroscientific research. Studies have reported improvements in cognitive functions [1, 2], motor processing [3], memory [4], and learning in healthy brains [5–7]. Additionally, tDCS has been studied in patients with neurodegenerative diseases, movement disorders, epilepsy, and poststroke language, attention, or executive deficits [8–13].

If shown to be reliable, tDCS may have several advantages that render it attractive for clinical use in comparison to invasive stimulation. The technique is, as stated, noninvasive and elicits only a slight tingling under the electrodes and can be applied continuously and safely for up to 20 minutes [14–17]. The device is also easy to use, small, and relatively inexpensive [5]. One area where noninvasive enhancement of neural function may be of benefit is Brain Computer Interfacing (BCI). In particular, its possible use in communication aids and in motor rehabilitation is considered in this paper.

BCI-based communication aids have been developed in the form of word spellers to help people with severe motor disabilities communicate with ease [18–20]. The concept of a BCI speller is based on a system that enables direct brain-to-character translation through the “oddball paradigm” [21]. However, P300 systems have had limited practical applications mostly because potential users may have reduced neural activity in one or multiple areas of the brain due to illness or damage. Ramaraju et al. [22] have looked at the effect of tDCS on P300 potentials and how tDCS may help facilitate better P300 responses. While not directly related, Antal et al. [23] reported measurable effects of tDCS on Visual Evoked Potentials (VEP) and Lee et al. [24] reported measurable effects of tDCS on latency and amplitude.

The other types of brain signals commonly used in BCI applications are the ones generated by motor imagery (MI). There too, potentially beneficial effects of tDCS have been reported such as in the rehabilitative motor training of patients who have suffered subacute strokes [25]. Results from a number of studies suggest that ERDs can be slightly and temporarily amplified to heighten responsiveness [16, 26–28]. tDCS has been reported to heighten the magnitude of alpha waves [29] and also be used in conjunction with BCI [30].

However, many studies that have shown a unidirectional change in the ERD level and an increase in ERD with A-tDCS did not use a tricombination of randomization of stimulations/cues, sham control, and a double-blind protocol as shown in Table 1. Thus, we set out in this work to measure the degree by which tDCS may elicit changes in ERD, ERS, and P300 using a robust experimental protocol which includes double-blinding, sham control, and randomization. Part of this work has been presented here were presented in a preliminary form as a conference paper [22].

## 2. Materials and Methods

### 2.1. Subject Selection

Ten right-handed subjects (aged 22 ± 3 years) participated in this study after giving written informed consent. No subject had any history of a neurological condition or had been receiving any acute or chronic medication affecting the central nervous system. This investigation has been given ethical approval by the University of South Wales.

### 2.2. A-tDCS Application Protocol

The tDCS device (HDCStim HS0023L02-73, Newronika S.r.l) with electrodes of size 5 cm × 5 cm was fitted according to the procedure used by DaSilva et al. [32] for anodal stimulation. The anodal electrode was placed over the left M1 and the cathodal electrode over the right supraorbital area. Subjects sat in an armless chair, and then using measurements of the distance between inion and nasion Cz was found, and then 20% of the distance from Cz to the left preauricular point was found. EEG high conductivity gel was then applied to this location, C3, and the temple. The tDCS pads were soaked in normal tap water, and high conductivity gel was applied. The pads were placed on C3 and right frontal lobe and both secured with a plastic band and netted cap.

Each subject underwent two experimental A-tDCS sessions, one real and one sham, separated by a one-week interval. The order of the A-tDCS presentation (sham or real) was randomized and double-blinded (neither the investigator nor the participants were made aware of what stimulation is being administered). Double-blinding was achieved by an independent investigator generating a sequence of random binary number pairs and associating them to real and sham in an undisclosed file. The A-tDCS option (real or sham) was selected for each participant by the independent investigator using the predetermined sequence: turning the sham button either “on” or “off” outside the view of the main researcher and the subject. The independent investigator did not interact with the experiment after applying the designated option. At the end of each session, the independent investigator verified that the researcher did not know whether the stimulation was applied to check the effectiveness of blinding. Furthermore, participants were not informed that the current intensity would be varied for each study (sham and real).

The real A-tDCS consisted of a 1.5 mA current applied for 15 minutes. The sham consisted of a dose of 1.5 mA ramping up from 0 mA to 1.5 mA over 10 s, followed by 8 s at 1.5 mA before the A-tDCS automatically turned off. This was done to mimic the transient skin sensation at the beginning of actual A-tDCS without producing any potential conditioning effects on the brain [33]. The complete study was run on each participant within one hour of removing the A-tDCS, which is within the time window reported previously for effects of tDCS to be detected [23, 34, 35].

The impedance value of the A-tDCS while operating was checked by the independent investigator and remained between 4 kΩ and 9 kΩ for all participants, which was the recommended window given in the A-tDCS device instructions.

### 2.3. EEG Measurement Protocol

After the application of real or sham stimulation, the EEG electrodes in the cap were connected, taking between 10 and 20 minutes to connect and apply conductive gel to the electrodes. Once connected, the impedance of each electrode remained at a maximum of 20 kΩ with a typical value being under 10 kΩ throughout the experiment. EEG signals were recorded from 14 Ag/AgCl disc electrodes (1 cm in diameter) with the ground at AFz and the reference electrodes at FCz. All the electrodes are placed according to the international 10–20 system. The signal was prefiltered (0.2–45 Hz) and a digital notch filter was applied at 50 Hz. The cap was centered on the scalp at Cz midway between the inion and nasion. Cz was checked for its equidistance to both left and right preauricular points.

Subjects sat in an armless chair with their eyes open facing a computer monitor placed approximately 0.7 m in front of them at eye level. Both arms dangled freely by their sides towards the ground. They were asked to avoid any further muscular activity including blinking. However, they were informed not to be concerned about accidental blinking or flinching. Participants were told that the study would be repeated several times and that occasional artefact would be removed. This was aimed at preventing stress in the participants if they did accidentally blink or move, the stress of which may have affected the remaining results.

### 2.4. ERD/ERS Measurement Protocol

Subjects were shown a tennis ball on the computer screen and asked to use their right hand in an attempt to grab it and then to let go and return their hand to its original position dangling freely by their side. They would perform this routine physically, clasping their hand within 1 cm of the screen before bringing it back. They were then asked to keep their arm and hand dangling freely by their side and mentally simulate the performance of the same reach-and-grasp motion, without moving either their arm or hand. Initially, during the rehearsal phase of this part of the study, the researcher held the participant's arm and shoulder gently to detect any muscular contractions. This rehearsal phase for each subject was conducted for both sham and tDCS sessions. Subjects were asked to repeat the imagined reach-and-grasp procedure until no muscular movement was detectable by the investigator. It was made clear to participants that they were required to make the mental effort to grab the ball in the same manner they had practiced physically and not just to imagine a video playback of their hand grasping it.

**Figure 1:**
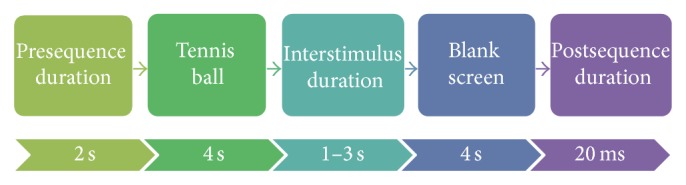
Cue timeline for ERD experiment.

Each trial consisted of two cues, a blank slide which appeared for 5 to 8 s (randomized) followed by a cue with a tennis ball centered on it. The blank cue signified the adoption of a rest state by the participant. When the tennis ball cue appeared, the participant was asked to make the mental effort of reaching out to the ball, grasping it, letting it go, and returning their arm to their side. These were all done with no time gaps in between each step. The presequence duration (blank-cue) appeared once at the start of the experiment. After this, the screen would display a tennis ball for 4 s followed by a blank screen. The blank screen was made up of three sequential blank slides. These three slides were used to allow for randomization in the length of time for which the blank black cue appeared before the tennis ball reappeared. They were made up of the interstimulus duration, the blank cue, and the postsequence duration. The interstimulus duration slide was on screen for between 1 and 3 seconds. All three blank slides appeared as one cue. From the perspective of the subject, they only saw two cues: a blank black cue and a tennis ball cue. A total of 6 trials per subject were conducted in each of the real and sham experiments totaling 120 trials overall. These steps are summarized in Figure 1.

All the EEG trials were visually inspected for any abnormal signals from the electrode contacts or muscular artifacts across C3 and C4 and these trials were omitted. C3 and C4 were selected as they produced the highest ERD/ERS response compared to all other channels. The EEG signal from C3 and C4 was filtered between 8 Hz and 13 Hz using a standard FIR filter. The epochs (−1 s to 3.5 s) were extracted, and baseline removal was run using the time window (−1 s to 0 s) on each channel's data. The epochs were averaged, and the power (*µ*V^2^) was found for the time windows before cue (−1 s to 0 s) and after cue (0.5 s to 3.5 s). The latter time window was selected to characterize the ERD signal from mentally reaching, clasping, and releasing the ball. The physical act took less than 3 seconds to complete. Both power values were normalized to the number of frequency points.

ERS values for C3 and C4 were arrived at by filtering the original preprocessed signal between 13 Hz and 24 Hz followed by baseline removal using the time window (−1 s to 0 s). The time segment 1 s to 5 s was used for analysis. All processing was done using EEGLAB [36]. For both cases of sham and real, ERD and ERS were calculated across both channels C3 and C4. The blank-cue power was termed the neutral power. The tennis ball cue power was termed the motor imagery power. The following measures were used to calculate the effect of A-tDCS on the ERD and ERS potentials:

(1) $$\begin{array}{c} RPD=P_{Neutral}-P_{Imagery}, \end{array}$$

where RPD stands for Relative Power Difference. *P*_Neutral_ is blank-cue power, and *P*_Imagery_ is tennis ball cue (active motor imagery) power measured at the ERD (8 Hz–13 Hz) and ERS (13 Hz–24 Hz) bands, respectively. Both power values were normalized to the number of frequency points.

### 2.5. P300 Measurement Protocol

A P300 oddball speller, which contains all characters (A–Z), numbers from 0 to 9, and spacebar, was presented to the volunteer in 6 × 6 matrix form [37]. The participant was asked to “spell” the nineteen (including spaces) letters in “THE QUICK FOX JUMPS” by focusing on the character inside the 6 × 6 matrix which they wanted to select. Two sequences were used to select a character. In a sequence, each row/column is intensified randomly. For each sequence, there are up to 12 intensifications (6 rows and 6 columns), and therefore a total of up to 24 intensifications are used to evoke a response to a character. The following measures were used to assess a P300 oddball response to intensified letters:

(2) APR=AveragePTarget,RPR=AveragePTarget−AveragePNon-targetAveragePTarget.

APR stands for “absolute P300 response” which considers only the P300 signal power in *µ*V^2^, whereas RPR stands for “relative P300 response” which considers the difference between the responses to target letters and nontarget letters.

EEG signal was measured for 19 target letters per subject per experiment totaling 380 EEG target samples. A larger number of “nontarget” samples were also measured (due to oddball experiment inherently generating more nontarget than target letters). These were used for RPR calculation.

*P*_Target_ is the average signal power in *µ*V^2^ between 250 ms and 450 ms of 19 intensified target letters. *P*_Non-target_ is the average signal power in *µ*V^2^ between 250 ms and 450 ms of all intensified nontarget letters.

## 3. Results and Discussion

### 3.1. Effect on ERD/ERS

**Figure 2:**
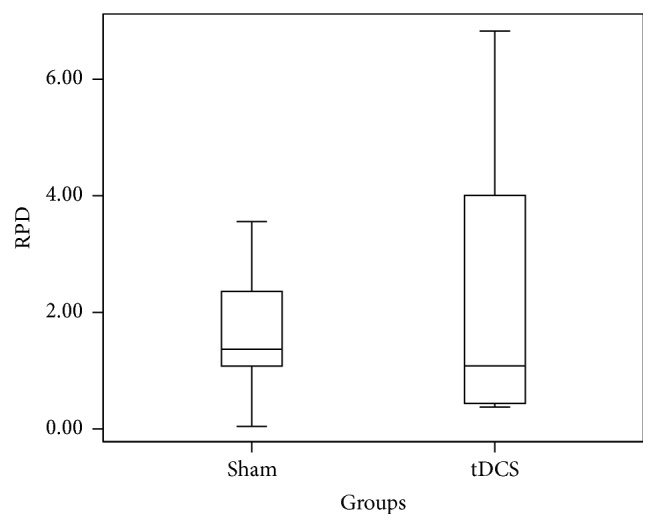
Box and whisker plot of the step in relative ERD power in *μ*V^2^ for C3 before and after motor imagery cue for the 10 participants.

**Figure 3:**
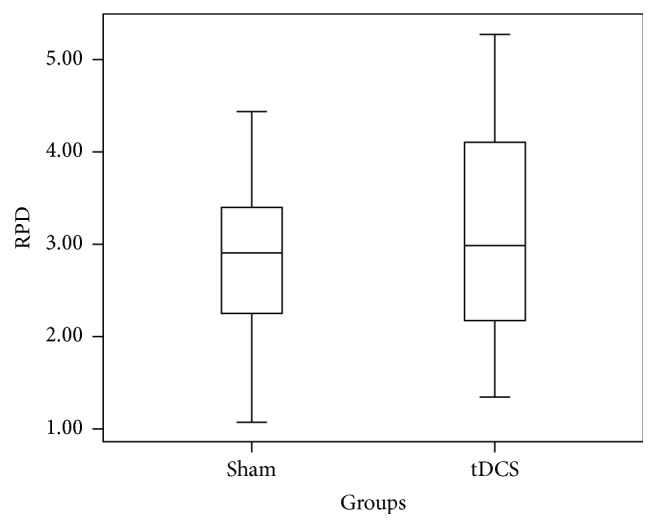
Box and whisker plot of the step in relative ERS power in *μ*V^2^ for C3 before and after motor imagery cue for the 10 participants.

Relative Power Difference (RPD) in ERD is measured for individual subjects across C3 for both real A-tDCS and sham A-tDCS conditions. A one-way ANOVA resulted in a* p* value of 0.46. The box plot in Figure 2 summarizes the data in the two groups. The overlap of two groups is clearly visible, and this supports the above* p* value. The same analysis was carried out for the RPD of ERS across C3. The box plot in Figure 3 summarizes the data in the two groups and clearly depicts overlap in the data from the two groups (real A-tDCS and sham A-tDCS). A one-way ANOVA test of mean difference yielded a* p* value of 0.49. The average power increment after application of the A-tDCS was 26.17%.

**Figure 4:**
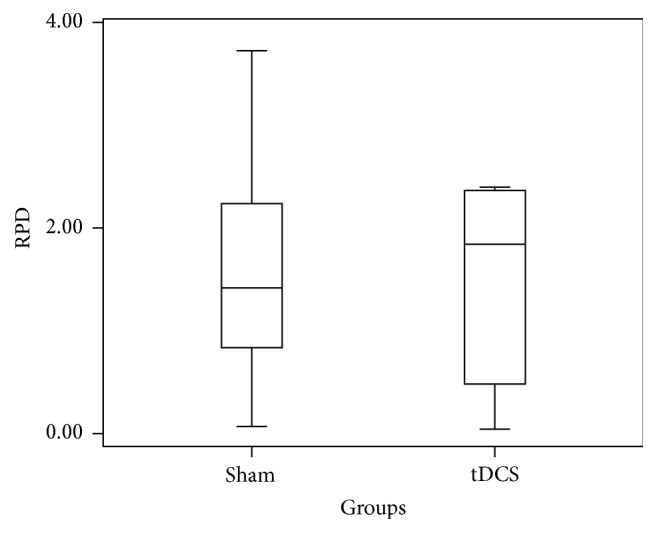
Box and whisker plot of the step in ERD power in *μ*V^2^ for C4 before and after motor imagery cue for the 10 participants.

**Figure 5:**
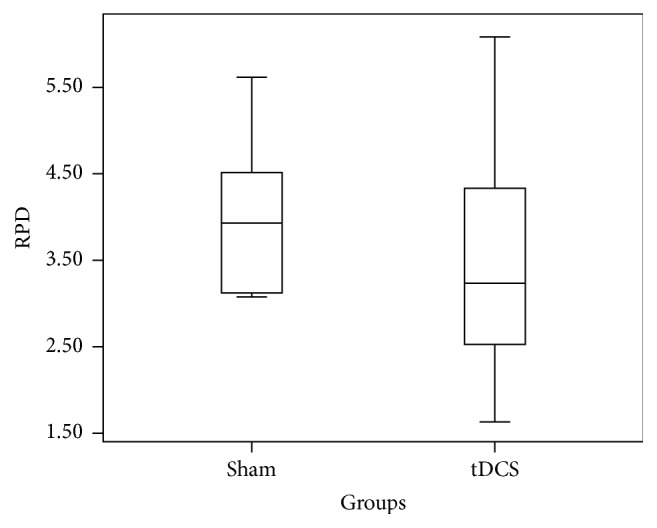
Box and whisker plot of the step in ERS power in *μ*V^2^ for C4 before and after motor imagery cue for the 10 participants.

The Relative Power Difference in ERD across channel C4 for both real and sham groups was measured and summarized in the box plot of Figure 4. The box plot clearly depicts the nonsignificance of difference (one-way ANOVA, *p* = 0.80) between sham and real groups. The average power change after the application of A-tDCS was 59%. The same analysis was performed on the ERS data from channel C4 (Figure 5) which clearly shows the overlap of the two groups (one-way ANOVA, *p* = 0.52) which gives the statistically insignificant difference between the two groups. In this case, the average power decrement after A-tDCS was found to be 10.39%. The Mu rhythm was then split into lower Mu (8 Hz–10 Hz) and upper Mu (10 Hz–13 Hz) and the RPD values in these two frequency bands across two channels C3 and C4 were calculated. One-way ANOVA results (*p* values: 0.98, 0.15, 0.78, and 0.337) clearly show the statistical insignificance in both lower and upper Mu.

### 3.2. Effect on P300

**Figure 6:**
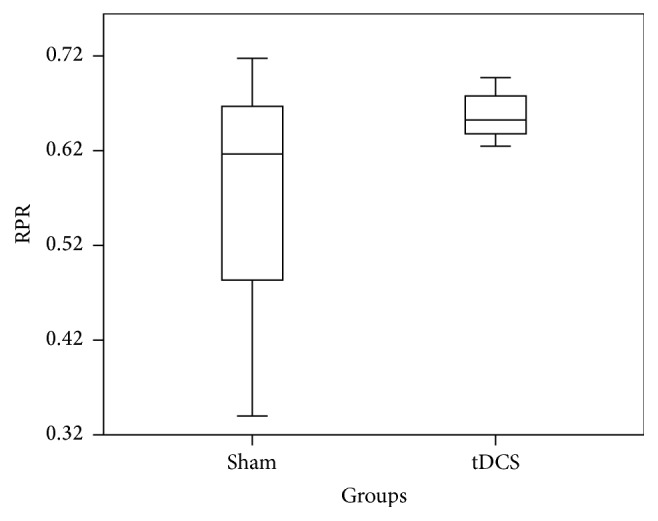
Box plot showing relative P300 response distribution of sham and tDCS across channel Oz for time window 250 ms–450 ms.

**Figure 7:**
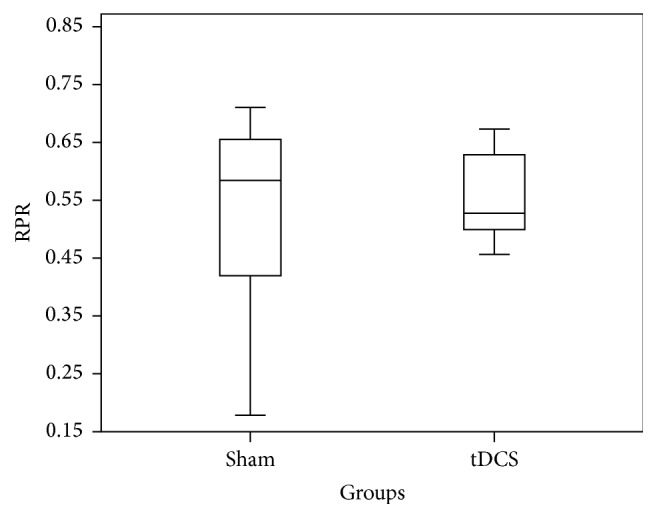
Box plot showing relative P300 response distribution of sham and tDCS across channel Pz for time window 250 ms–450 ms.

**Figure 8:**
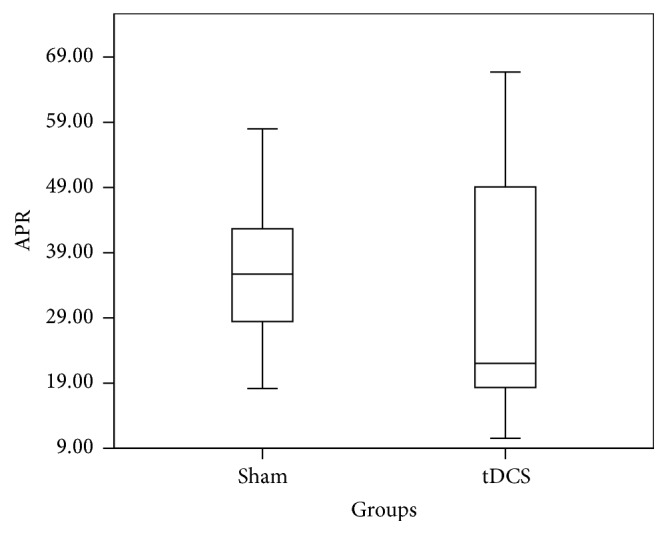
Box plot showing absolute P300 response in *μ*V^2^ distribution of sham and tDCS across channel Oz for time window 250 ms–450 ms.

The average change in the RPR across channel Oz for all subjects was a 22% increase following A-tDCS when compared to sham (data for subjects 9 and 10 was corrupt and thus not included). The box plot in Figure 6 depicts RPR values of both groups. From Figure 6, there is no clear separation of sham and A-tDCS groups. The real group appears to have a smaller standard deviation than that of the sham group (sham: 0.13, A-tDCS: 0.07). The absolute value of P300 however across Oz is not significantly different between groups (Figure 8) with a* p* value of 0.42. The same analysis was carried out on the data from channel Pz resulting in a* p* value of 0.578, which suggests a statistically insignificant difference between the t-DCS and the sham groups. The RPR data across Pz is summarized in a box plot in Figure 7. We note that the real group has a lower standard deviation (0.076) when compared to the sham group (0.176), though this difference may not be significant. The power at every time instant across all the subjects was averaged for both the sham and the real groups.

**Figure 9:**
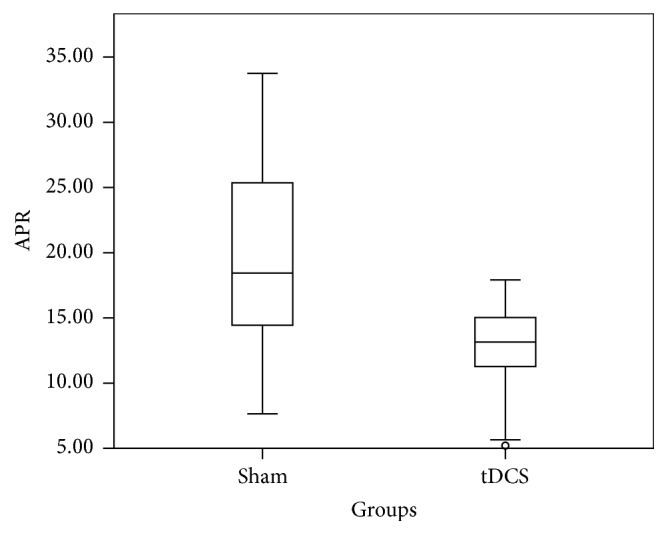
Box plot showing absolute P300 response in *µ*V^2^ distribution of sham and tDCS across Pz channel for time window 250 ms–450 ms.

**Figure 10:**
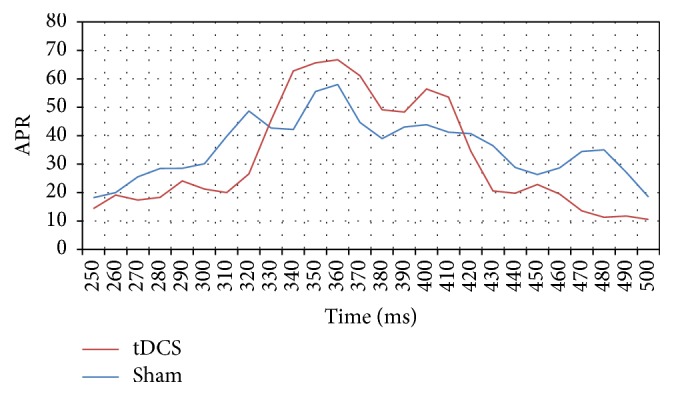
Graph of absolute P300 response in *µ*V^2^ of 8 subjects across channel Oz for tDCS and sham conditions for time window 250 ms–450 ms.

**Figure 11:**
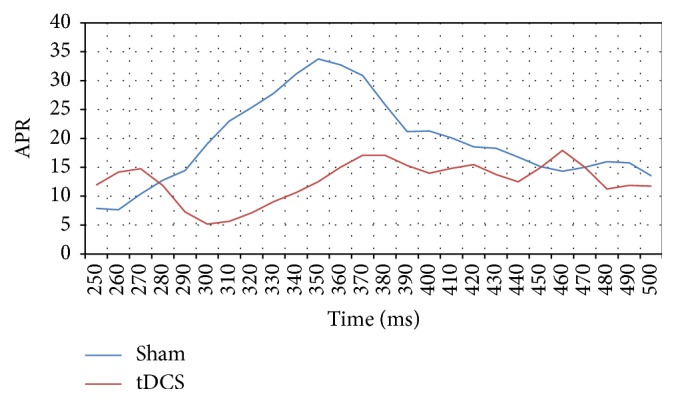
Graph of absolute P300 response in *µ*V^2^ of 8 subjects across Pz channel for tDCS and sham conditions for time window 250 ms–450 ms.

The absolute P300 response for channel Oz is summarized in Figure 10. Although there appears to be a high APR for the real group when compared to the sham group between 314 ms and 380 ms, a one-way ANOVA performed on the APR data from each group indicates a statistically insignificant difference between the groups (*p* = 0.42). A similar analysis was performed across the APR data for channel Pz (depicted in Figure 11) and gives a clear difference in the APR between 270 ms and 400 ms where major differences around 300 ms can be observed (one-way ANOVA with a* p* value 0.0002, Figure 9). All the averages and standard deviations across C3, C4, Pz, and Oz for sham and tDCS are summarized in Table 2. Although ANOVA is robust under an equal variance assumption, we have nevertheless confirmed the findings with the nonparametric Kruskal-Wallis test which yielded nearly similar* p* values.

## 4. Conclusions

Our results show that A-tDCS has had a significant effect on the absolute P300 response. This may help the development of neurorehabilitation methods targeting the parietal lobe. Heightening of the P300 response using A-tDCS may also help improve the accuracy of P300 based oddball paradigm spellers for neurologically impaired subjects. These spellers, although they have been shown to work in principle, have had limited practical applications partly because potential users often have reduced neural activity in one or multiple areas of the brain due to illness or damage. A rehabilitation regime of A-tDCS stimulation, used in conjunction with oddball paradigm spellers, could improve their usability, hence benefiting their users by allowing them to communicate. These users primarily include locked-in syndrome sufferers from conditions such as motor neuron disease (MND), stroke, and traumatic brain injury.

On the other hand, our study also demonstrated that the A-tDCS had no effect on ERD/ERS responses during motor tasks. This presents a complex picture of the effect of tDCS in general, as it may be specific to brain areas and functions. This appears to be consistent with a number of studies that when taken together exhibit varied findings on the effects of A-tDCS on EEG measurable cortical activity [38–40].

It may be the case that the lack of double-blinding in a number of earlier positive studies may have played a role in their results (Table 1) since failing to double-blind a study may allow for the invigilator to influence the subject as to the dosage given. There is also the possibility of a progressive training element over time as familiarity increases with the sequencing. One possible factor affecting A-tDCS effect on ERD-ERS might be that competing mechanisms of inhibitory and executory mechanisms may be at play when A-tDCS is operational, thus not allowing for a consistent outcome. The outcomes in our study may also be because the execution or imagination of the arm and hand movement occurs within the great functional and anatomical complexity of the Supplementary Motor Area (SMA) and its somatotopic organization in the form of a pure motor area and a mixed sensorimotor area [41]. The orientation and pathways may have a bearing on the outcome as has been reported in another study that found unexpected results from tDCS [34]. The authors of [34] took the standpoint that so far it has not been shown whether the excitability changes resulting from tDCS of the frontal cortex or even subcortical stimulation are similar to those induced by motor cortical tDCS. Participant genotype may also be a factor, as a study failed to demonstrate MEP facilitation after A-tDCS in one group carrying a specific genotype with only a hint of early facilitation which was not statistically significant [42].

The positive P300 effect observed might also indicate that the A-tDCS effect is not localized, which is in line with findings of widespread activation in several brain regions [33]. And, hence, A-tDCS might have more effects on nonlocalized EEG patterns such as P300 and fewer effects on localized EEG pattern such as motor related ERD/ERS. This hypothesis would need further research to be tested using combined fMRI and tDCS studies, for instance, where the effect of stimulation on the activation of particular cortical regions can be studied. What is evident is that A-tDCS can have an effect on some brain potentials and not others and that this complex picture can only be understood with robust and well-controlled studies.

Future work will also focus on the effect of secondary factors such as age, gender, and psychometric profiles as well as using computer simulation of current flows combined with subject imaging data to work out optimal electrode placement for desired applications. An interesting question would be whether replicating the same montage in two morphologically different individuals reproduces the same stimulation patterns. This can be answered with detailed subject specific and anatomically accurate computer simulation of current flows in the cortical areas of the brain. This then could lead to individualized imaging driven montages which may be a better way of conducting controlled studies in all targets of tDCS.

Finally, because the efficacy of A-tDCS is still a debated topic in the literature, it is unlikely that one paper will provide a definitive answer for all aspects of A-tDCS impact on EEG. As in many scientific controversies, systematic studies need to be done looking at the literature for a consensus to be built. It is therefore important that robustly controlled studies are carried out and that positive, as well as negative, studies are duly reported.

## Figures and Tables

**Table 1:** List of papers which did not use randomized, double-blind, and sham-controlled protocols in their research.

Paper	Year	Randomized	Double-blind	Sham-controlled	*p* value
Kasashima et al. [31]	2012	Yes	No	No	0.018
Matsumoto et al. [7]	2010	Yes	No	No	0.001
Wei et al. [6]	2013	Yes	No	Yes	0.023
Notturno et al. [16]	2014	Yes	No	No	0.035
Roy et al. [17]	2014	Yes	No	Yes	0.001
Nitsche et al. [3]	2003	Yes	No	Yes	0.001
Lee et al. [24]	2014	No	No	No	<0.05

**Table 2:** Summary of experimental results.

Power measure		Sham				tDCS				ANOVA	
		Oz		Pz		Oz		Pz			
		Average	Standard deviation	Average	Standard deviation	Average	Standard deviation	Average	Standard deviation	Oz	Pz
P300	Relative	0.573	0.13	0.526	0.176	0.666	0.07	0.553	0.076	**0.103**	0.578
	Absolute	35.81	10.54	19.56	7.41	32.08	19.31	12.61	3.45	0.422	**0.0002**

		C3		C4		C3		C4		C3	C4

Motor imagery	ERD	1.59	1.003	1.64	1.25	2.16	2.19	1.82	1.73	0.46	0.80
	ERS	2.98	1.288	3.84	1.36	3.49	1.93	3.44	1.36	0.49	0.52
